# Scorer and modality agreement for the detection of intervertebral disc calcification in Dachshunds

**DOI:** 10.1186/s13028-018-0416-2

**Published:** 2018-10-13

**Authors:** Alana Jayne Rosenblatt, Anu Katriina Lappalainen, Nina Alice James, Natalie Siu Ling Webster, Charles Grégoire Bénédict Caraguel

**Affiliations:** 1 School of Animal and Veterinary Sciences, The University of Adelaide, Roseworthy Campus, Adelaide, SA 5005 Australia; 2 Faculty of Veterinary Medicine, University of Helsinki, Viikki Campus, Agnes Sjöberginkatu 2, 00014 Helsinki, Finland; 3 Darkroom Veterinary Imaging, 7/489A Warrigal Rd, Moorabbin, VIC 3189 Australia; 4 Present Address: School of Veterinary Science, The University of Queensland, Gatton, QLD 4343 Australia

**Keywords:** CT, Dachshund, Intervertebral disc calcification, MRI, Radiography, Repeatability, Reproducibility, Scoring

## Abstract

**Background:**

The Dachshund is a chondrodystrophic breed of dog predisposed to premature degeneration and calcification, and subsequent herniation, of intervertebral discs (IVDs). This condition is heritable in Dachshunds and breeding candidates are screened for radiographically detectable intervertebral disc calcification (RDIDC), a feature of advanced disc degeneration and a prognostic factor for clinical disease. RDIDC scoring has been previously shown to be consistent within scorers; however, strong scorer effect (subjectivity) was also reported. The aim of this study was to estimate the within- and between-scorer agreement (repeatability and reproducibility, respectively) of computed tomography (CT) scanning and magnetic resonance imaging (MRI) for scoring IVD calcification, and to compare these modalities with radiographic scoring.

**Results:**

Twenty-one Dachshund dogs were screened for IVD calcification using the three imaging modalities. Three scorers scored each case twice, independently. Repeatability was highest for radiography (95.4%), and significantly higher than for CT (90.4%) but not MRI (93.8%). Reproducibility was also highest for radiography (92.9%), but not significantly higher than for CT or MRI (89.4% and 86.4%, respectively). Overall, CT scored IVDs differently than radiography and MRI (64.8% and 62.7% agreement, respectively), while radiography and MRI scored more similarly (85.7% agreement).

**Conclusions:**

Despite high precision for radiography, previous evidence of scorer subjectivity was confirmed, which was not generally observed with CT and MRI. The increased consistency of radiography may be related to prior scorer experience with the modality and RDIDC scoring. This study does not support replacing radiography with CT or MRI to screen for heritable IVD calcification in breeding Dachshunds; however, evaluation of dog-level precision and the accuracy of each modality is recommended.

## Background

Of all the dog breeds, the Dachshund has the highest lifetime incidence of intervertebral disc disease (IVDD) [1, 2]. The results of a recent study in the UK, based on a survey of Dachshund owners (“Dachs-Life 2015”), found an overall IVDD prevalence of 15.7% in the surveyed Dachshund population of 1975 dogs, with a significant prevalence range between different breed variants (7.1–24.4%) [3]. This high prevalence may be due to a variety of genetic, physical and lifestyle-related factors [3], but is likely primarily attributable to their chondrodystrophic morphology. Dogs with chondrodystrophy undergo chrondroid metaplasia, the premature maturation and degeneration of intervertebral discs (IVDs) that often results in calcification, an indicator of severe degeneration [2, 4, 5]. These degenerated IVDs are predisposed to herniate into the spinal canal under minimal stress, resulting in spinal cord compression and injury [4]. Dachshunds with IVD herniation have a high level of morbidity and mortality, and despite treatment that often includes complex and costly surgical intervention, a substantial proportion of dogs retain neurologic deficits [6–8]. IVDD is widely accepted as the Dachshund breed’s greatest health problem.

A scheme for radiographic scoring of intervertebral disc calcification in Dachshunds has been developed, and recently reviewed [9]. Radiographically detectable intervertebral disc calcification (RDIDC) is highly heritable in Dachshunds [10–14], and the development of RDIDC at a young adult age corresponds with an increased risk of developing clinical IVDD during the lifetime of the dog [8, 10, 15–18]. Therefore, screening young adult breeding candidates for RDIDC, ideally at 24–30 months of age, can reduce the prevalence of the disease in the breed [11, 18, 19]. RDIDC is scored from 0 to a maximum of 26 (i.e. 26 total IVDs in the canine cervical, thoracic and lumbar spine). Current screening programs recommend that Dachshunds with RDIDC scores of ≤ 2 are suitable for breeding, dogs with scores of 3–4 should be bred judiciously, and animals with scores ≥ 5 should be excluded for breeding purposes [8, 11, 12, 17, 18].

For a screening test to be useful in a selective breeding program, it must be precise (i.e. very reproducible). Recent evaluation of within- and between-scorer agreement for RDIDC scoring identified an overall high level of repeatability and reproducibility, but also identified some limitations of radiography as a screening tool [20]. Test precision was influenced by scorer experience level (expert scorer > specialist radiologist > general practitioner), which in turn affected the consistency (agreement) of the results. Individual scorer-dependent subjectivity was also identified.

The absence of RDIDC does not exclude a disc from being degenerative nor calcified, and only a portion of IVD calcifications present in a spine would be expected to be detected radiographically [17, 21]. It is postulated that a cross-sectional imaging modality such as computed tomography (CT) scanning would be a superior alternative for screening dogs for IVD calcification compared to radiography, as CT reduces challenges associated with anatomic superimposition and has improved contrast resolution [22, 23]. Alternatively, magnetic resonance imaging (MRI) is a cross-sectional modality with superior contrast resolution to both CT and radiography, and high-field MRI is considered the optimal modality for imaging the spine [24, 25]. MRI of intervertebral discs allows identification of earlier stages of disc degeneration than calcification, due to its ability to detect biochemical changes in tissues including loss of water and proteoglycan content and decreased chondroitin-keratan sulfate ratio in the nucleus pulposus, such that both degenerative and calcified IVDs have decreased MR signal intensity [22, 26–29]. That is, MRI detects a spectrum of IVD degeneration but cannot differentiate between calcified and non-calcified degenerative discs, compared to radiography and CT which can only detect disc calcification as an indicator of (advanced) degeneration. IVD degeneration in the canine spine can be reliably graded using low-field MRI and the Pfirrmann classification system, which is based on lumbar IVD degeneration in people and has been verified with the gross pathology-based Thompson system [30–33].

The precision of CT and MRI scoring of IVD calcification in Dachshunds has not been assessed. Thus, the objectives of this study were to: (i) compare the precision of three diagnostic imaging modalities (radiography, CT and MRI) by estimating their repeatability and reproducibility, (ii) estimate and compare the robustness (i.e. scorer independence) of each modality, and (iii) estimate the agreement across the three modalities for the detection of IVD calcification. It was anticipated that both CT and MRI would be more precise than radiography due to the cross-sectional nature of these modalities. However, it was expected that MRI would not completely agree with the two other modalities because this modality assesses various stages of IVD degeneration, not only calcification.

## Methods

### Study subjects

Dogs were prospectively recruited from Finnish Dachshund breeders through The Dachshund Club of Finland, between 22 November 2011 and 7 March 2012. Eligibility criteria included: purebred registered Standard Dachshund dog, young adult age (24–48 months old), and clinically healthy. Dogs were excluded if they had prior or current signs of intervertebral disc disease (IVDD) or other illness. Dogs were enrolled in the study with informed owner consent and the study was approved and conducted with animal ethics approval.

#### Diagnostic imaging

**Fig. 1:**
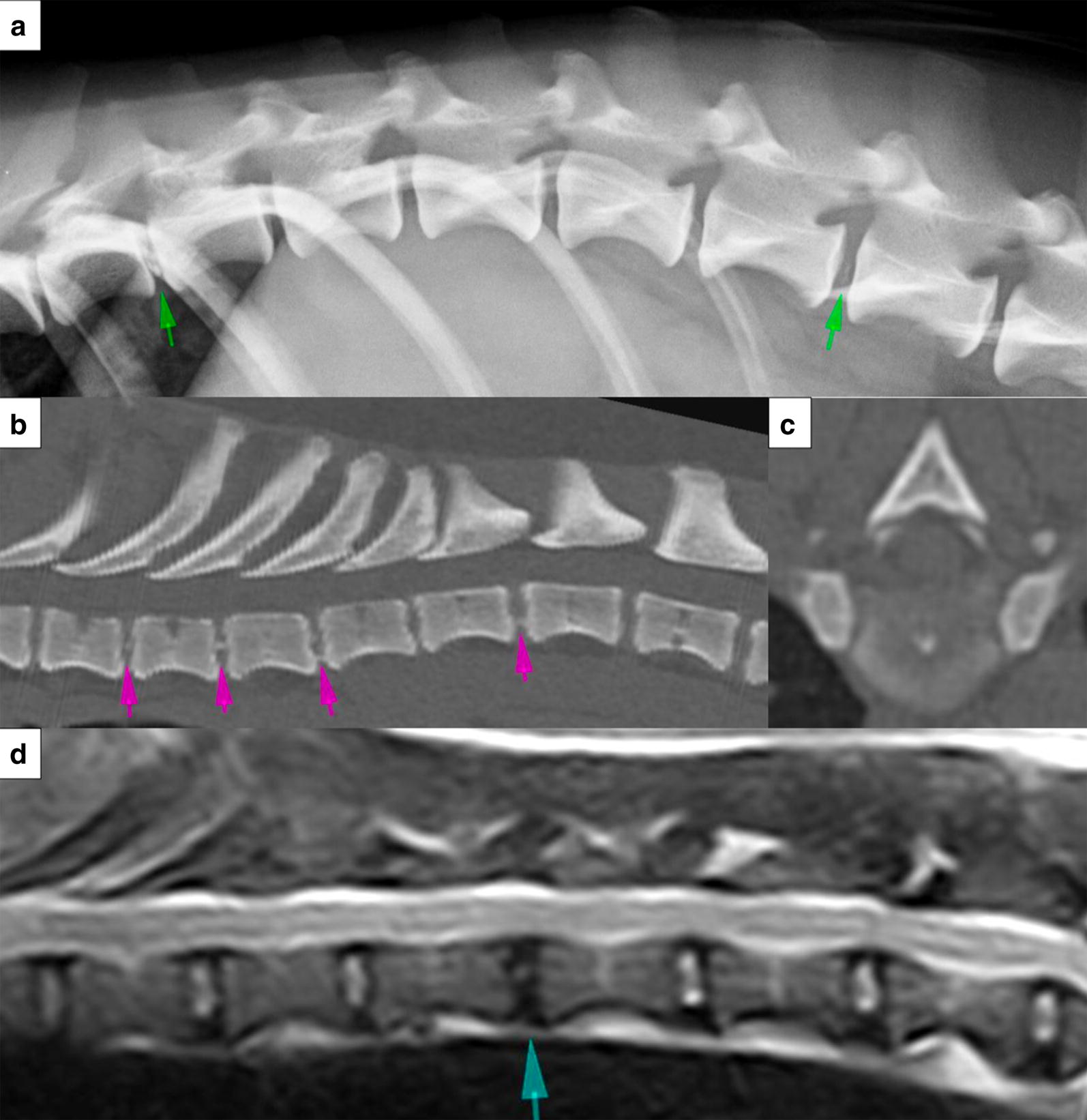
Example radiographic (**a**), CT (**b**, **c**) and MR (**d**) images obtained for intervertebral disc (IVD) scoring (not necessarily from the same Dachshund). The images are centered on the caudal thoracic spine. Example intervertebral disc calcifications are indicated on the lateral spinal radiograph (**a**; green arrows), and on the sagittal (**b**; pink arrows) and transverse (**c**) CT images which are displayed in a bone window. On the T2W sagittal MR image (**d**), the blue arrow indicates an MRI Pfirrmann grade 3 degenerative IVD. *CT* computed tomography, *MRI* magnetic resonance imaging

The imaging was performed at the University of Helsinki Veterinary Teaching Hospital. Three diagnostic imaging modalities were employed to image the dogs’ spines—radiography, CT scanning and low-field MRI (Fig. 1). All imaging was performed within a single hospital visit, with the dogs under heavy sedation or general anaesthesia. Radiography and CT were conducted on all dogs, while MRI was optional and based on owner preference given it would substantially prolong anaesthetic time for an elective procedure.

### Radiography

Lateral radiographs of the cervical, thoracic and lumbar spine regions were obtained for each dog using a previously described protocol [20] and a digital radiographic system (CPI Indico 100, Ontario, Canada). A minimum of five diagnostic quality radiographs were acquired for each dog.

### Computed tomography

CT was performed using a 2-slice helical scanner (Siemens Somatom Emotion Duo, Forchheim, Germany) with the following scan parameters: 100 mA, 110 kV, 1.0 mm acquisition slice thickness, feed/rotation 2 mm, rotation time 0.8 s, reconstruction interval 0.5 mm, bone algorithm (WL, 500; WW, 3500). CT scanner limitations (i.e. excess tube heat) did not allow for scanning of the entire spine. The thoracolumbar spine was of greatest interest due to the propensity for clinical IVDD in this region. Therefore, T5-L7 (or a portion thereof) was scanned in all dogs. Where possible, the cervicothoracic (C6-T2) and/or the lumbosacral (L7-S1) spine junctions were also scanned; these regions were selected as they are anecdotally challenging to score radiographically for IVD calcification due to issues with superimposition of anatomy.

### Magnetic resonance imaging

MRI studies of the thoracolumbar spine were obtained using a low-field scanner (Vet-MR 0.23T, Esaote S.p.A, Genoa, Italy) and the following pulse sequences: sagittal plane T1W (TR, 510; TE, 18), sagittal plane T2W (TR, 2800; TE, 80), and transverse plane T1W (TR, 830; TE, 18). As with the CT imaging, the limitations of using a low-field magnet (specifically, acquisition time) did not allow for imaging of the entire spine, so the thoracolumbar spine (T5-S1, or part thereof) was scanned, being the region of greatest clinical interest.

### Scoring

Three veterinarians who all had diagnostic imaging backgrounds and training, but varying levels of RDIDC scoring experience, performed the scoring of the intervertebral discs. All cases were duplicated, coded (with individual identifying information removed from the images), and randomly ordered prior to distribution to ensure blinding of the scorers. The imaging studies were viewed in Digital Imaging and Communications in Medicine (DICOM) format using OsiriX image viewing software (Pixmeo, Geneva, Switzerland) and high resolution/brightness, commercial-grade monitors, with freedom to post-process images as preferred by the individual.

Each radiographic study was scored for the presence or absence of IVD calcification. The CT cases were distributed 1 month after the radiographic scoring had been completed to facilitate scorer blinding. The subjective presence or absence of IVD calcification was recorded, as was scorer confidence in their decision and approximate percentage of calcification of the total disc cross-sectional area (in 10% increments, 0–100%). Again, MRI cases were distributed 1 month after all scorers had completed the CT scoring. Based primarily on the sagittal T2W images [32], IVDs were graded for any sign of degeneration (i.e. not specifically calcification) following the Pfirrmann classification scheme [30, 33], which uses visual analysis of the IVD structure, distinction between nucleus pulposus and annulus fibrosis, MR signal intensity, and height of the IVD, to grade a disc on a scale of 1 (normal) to 5 (severe degeneration). Scorers were provided with example images and written description of the characteristics of each grade, as a reference. The scorers recorded results for each imaging study using custom scoring templates, as per a previous study [20]. Scoring decisions were made by independent opinion. Observers were aware that the dogs were clinically healthy but were otherwise blinded to patient details and other identifiers.

### Statistical analysis

Scores were collected, collated and formatted using Microsoft Excel (Microsoft Corporation, Redmond, WA, USA). An IVD score was classified as positive for calcification when calcification (≥ 10% of IVD area) was observed (radiographs and CT) or when the Pfirrmann grade was ≥ 3 (MRI), and classified as negative otherwise. Analyses for study objectives (i) and (iii) were conducted using the statistical package Stata version 14.2 (Stata Corp, College Station, TX, USA), and analysis for objective (ii) was conducted using the phylogenetic package MEGA version 7 [34].

### Modalities’ precision (repeatability and reproducibility)

Precision was evaluated by estimating the repeatability and reproducibility of the three modalities. For a given modality, repeatability was estimated as the proportion of pairs of scores that agreed within a given scorer. The reproducibility was measured as the proportion of pairs of scores that agreed between two scorers. To compare precision across modalities, separate datasets and logistic models were developed for repeatability and reproducibility. The datasets were reformatted in a long format with each observation reporting an agreement (coded as “1”) or a disagreement (coded as “0”) between two scorer iterations for a given dog’s IVD from a same scorer (repeatability dataset) or from two separate scorers (reproducibility dataset) of a given modality. Covariate factors included dog, IVD, modality, and scorer for each observation. Given that agreement observations were clustered within IVDs and IVDs were clustered within dogs, random effects for dog and IVD were added to the models to account for the lack of independence across observations. Also, given that the study dogs and their IVDs were scored up to 6 times by a same scorer (clustered within scorers), scorer was included as a random effect cross-classified with dog and IVD. When modeling reproducibility, models with cross-classified structure could not converge and the reproducibility was modeled using scorers’ pair, dog, and IVD random effect without cross-classification. Repeatability and reproducibility across modalities were estimated and compared by including modality as a fixed effect in the respective models.

The direct interpretation of the models’ coefficients (intercepts and/or effect coefficient), ignoring random effects, provides cluster-specific estimates of agreement. To obtain average estimates across dogs, scorers and IVDs (i.e. population-averaged interpretation), cluster-specific predicted agreements and the limits of the 95% confidence interval were converted to population-averaged values using the following approximation formula [35]:

1 $$Prob\left( {agreement} \right) \, \approx \, logit^{ - 1} \left( {\left( {\beta_{0} + \beta_{1} Modality} \right)/\surd \left( {1 + 0.346*\left( {\sigma^{2}_{scorer} + \sigma^{2}_{dog} + \, \sigma^{2}_{IVD} } \right)} \right)} \right)$$

where *β*_*0*_ is the model intercept coefficient; *β*_*1*_ *Modality* is the modality fixed effect (radiography set as default category); σ_scorer_^2^, σ_dog_^2^ and σ_IVD_^2^ are the scorer, dog and IVD within dog random effect variance, respectively; and logit^−1^ is the inverse of the logit function (*logit*^−1^*(x)* = 1/(1+*e*^−*x*^)). Post-regression inferences were two-sided and adjusted using the Bonferroni method (alpha, set at 5%, divided by the number of pairwise comparisons between modalities, alpha_Bonferroni_ = 1.7%).

### Modalities’ robustness (scorer independence)

The ruggedness of a test is defined as the capacity of the test to resist expected variation across users [36]. In other words, ruggedness measures how dependent the outcome of the test is on the person running or interpreting the test. Here, the ruggedness of each modality was investigated by determining the existence of scorer subjectivity when interpreting IVDs using a diagnostic imaging test. Similar to a previous report [20] and following the principle of a cluster analysis, distance-based Neighbor-Joining phylograms were built from an alignment of IVD scores (IVDs in columns and scoring iterations in rows) to identify the presence of iteration cluster(s) corresponding to distinct scoring patterns. If the two scoring iterations from a same scorer cluster together, there is evidence that the scoring from this scorer is distinct from the other scorers. To assess the robustness of the node linking two iterations together, bootstrap support values (proportion of resampled trees that include the node of interest) were generated using bootstrap-resampling 1000 times and reported as a percentage on the nodes of the original tree [37]. A node with a bootstrap support value of ≥ 70% was considered robust. The advantage of this approach is that it accounts for both the quantitative distance and the qualitative pattern across scoring iterations.

### Agreement across modalities

Agreement across modalities was estimated as the proportion of pairs of scores between modalities’ iterations that agreed within a given scorer. Comparisons between scorer iterations were ignored to exclude between-scorer effect. The same data structure, model building, and population-averaged interpretation as for repeatability and reproducibility were used. Agreement across modalities was explored across all MRI Pfirrmann grade cut-offs (i.e. ≥ 1 to = 5).

## Results

### Study subjects

Twenty-one young adult (age range, 26–45 months; median, 30 months; SD, 4.8 months) Dachshund dogs were recruited. The study population was relatively homogeneous, with dogs being intact females (n = 10), intact males (9), neutered female (1) and neutered male (1); breed variants being standard long-haired (11) or standard wire-haired (10); and dogs weighing 7.6–12.6 kg (mean, 9.8 kg; SD, 1.3 kg).

### Precision and robustness of each modality

**Fig. 2:**
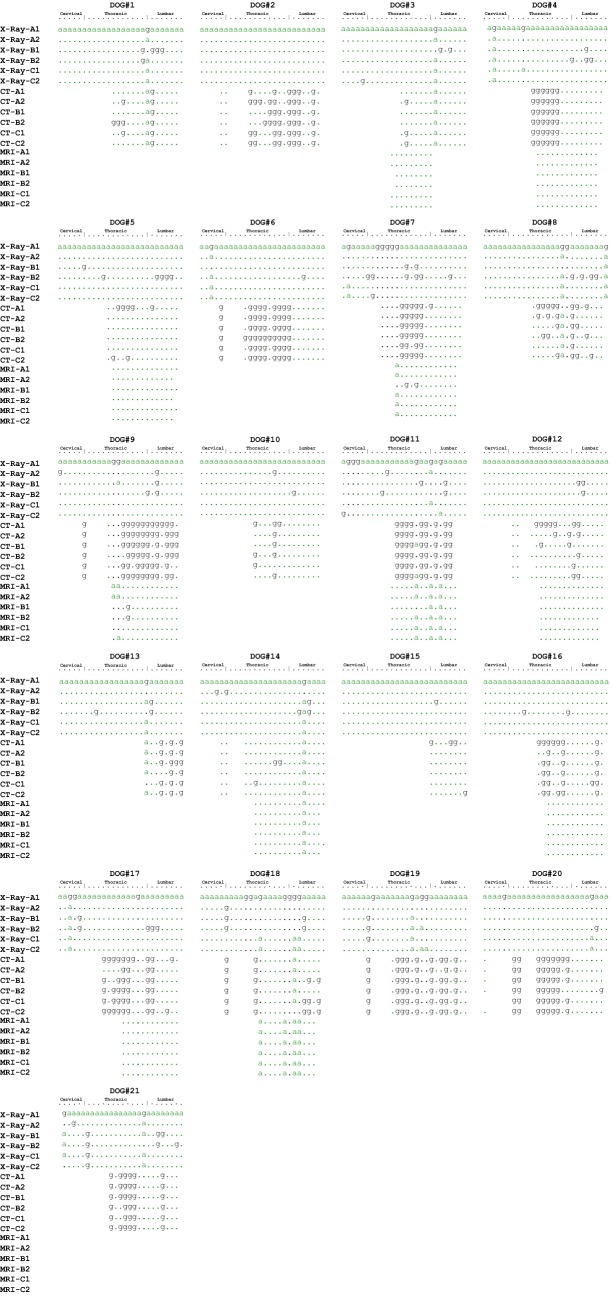
Scoring alignment of individual intervertebral discs (IVDs) scored (column) by each scorer (A, B and C) for each iteration (1 and 2) and each modality (X-ray, CT and MRI) (row). The intervertebral discs (IVDs) of each of the 21 participating Dachshund dogs are ordered per their location in the vertebral column i.e. position 1 (C2-3) to 26 (L7-S1). An “a” codes for a negative score, a “g” codes for a positive score, a “dot” codes for a score that agrees with the first row (X-ray iteration 1 of scorer A), and a “blank” codes for an absent IVD score due to missing data. “X-ray” denotes radiography; “CT” denotes computed tomography; “MRI” denotes magnetic resonance imaging

A summary of the score for each available IVD in each dog, for each scorer, each iteration and each modality, is presented in Fig. 2. Estimates and 95% confidence intervals (95% CI) of repeatability (within-scorer agreement) and reproducibility (between-scorer agreement) are reported (Table 1).

**Table 1:** Imaging modalities’ repeatability and reproducibility

Modality	Repeatability (95% CI)	Reproducibility (95% CI)
Radiography	95.4%^b^ (92.4–97.3)	92.9%^c^ (67.8–98.8)
CT	90.4%^a^ (84.8–94.1)	89.4%^c^ (62.8–97.7)
MRI	93.8%^a,b^ (88.9–96.6)	86.4%^c^ (60.4–96.4)

Model estimates of the repeatability and reproducibility for intervertebral disc (IVD) calcification scoring by radiography, computed tomography (CT) and magnetic resonance imaging (MRI) (interpreted as positive if MRI Pfirrmann grade ≥3), with 95% confidence intervals reported in brackets. Within a column, different superscript letters indicate significant differences between modalities

### Radiography

**Fig. 3:**
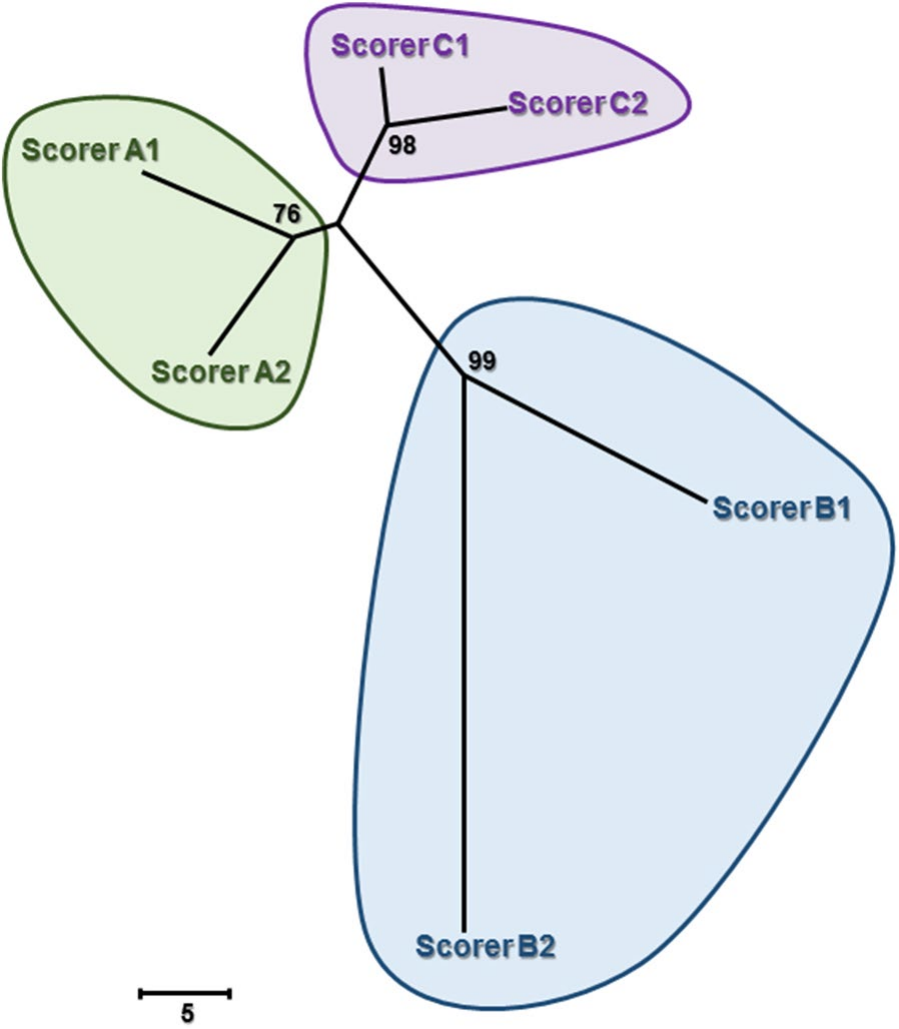
Phylogram demonstrating the agreement within and between scorers for radiographic scoring of intervertebral disc (IVD) calcification in 21 Dachshunds. The length of the branches between different scorers (A, B, C) represent the disagreement between scorers. The length of the branches between two scorer iterations (1, 2) represents the within-scorer disagreement. The scale is based on the number of differing scores out of the 544 IVDs assessed by an individual scorer. Numerical bootstrap values indicate strength. Scale bar = 5 IVD scoring differences

Except for the C2-3 IVD of dogs #4 and #21 (Fig. 2), all 26 potential IVDs from the 21 participating dogs (544 IVDs in total) were examined radiographically by each of the three scorers, two times independently (total, 3264 scores). The repeatability of radiography was slightly higher than its reproducibility suggesting at first little scorer effect (Table 1). However, the phylogram (distance tree) of IVD scoring using radiography identified three clear clusters, corresponding to each individual scorer, supported by high bootstrap values (> 70%) (Fig. 3). This revealed that each scorer had a scoring pattern that was unique enough to be discriminated from the other scorers. The length of the branches between two iterations reflects the amount of disagreement between these iterations (i.e. the shorter the branch length, the stronger the agreement between two iterations). Within each scorer, the distance between the iterations of scorer B were clearly longer than for scorers A and C, showing a lower repeatability for scorer B. Across scorers, scorer B was further away from the other two scorers corresponding to poorer reproducibility for this scorer.

### Computed tomography

**Fig. 4:**
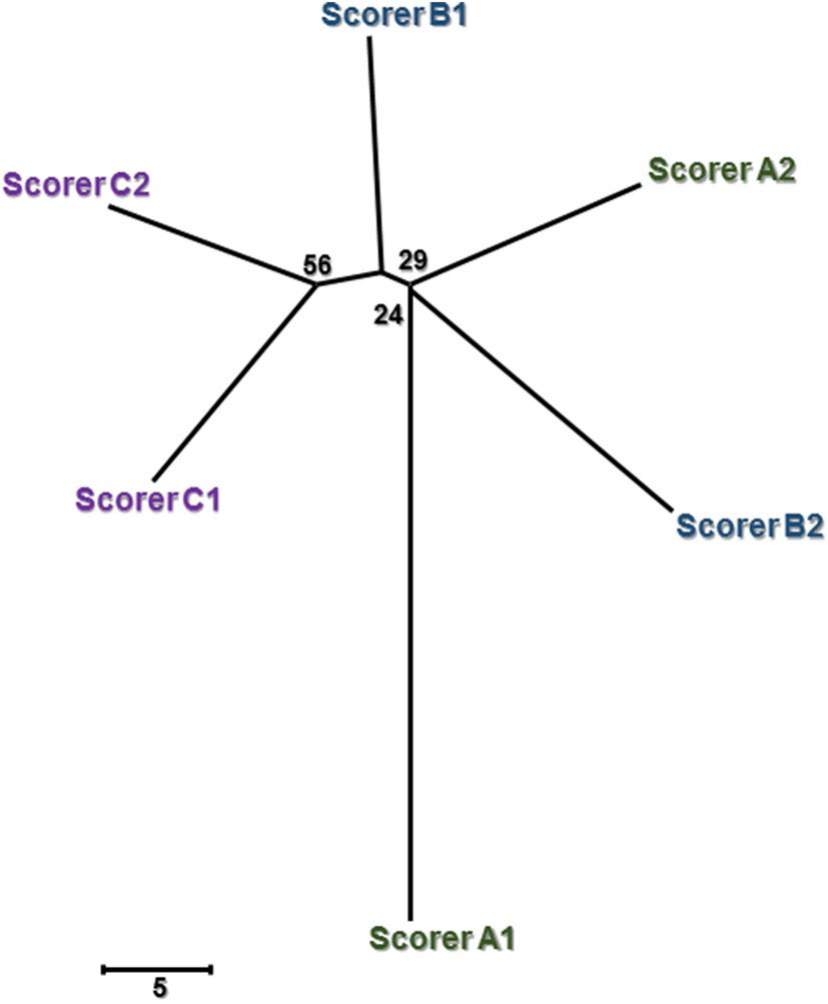
Phylogram demonstrating the agreement within and between scorers for computed tomographic (CT) scoring of intervertebral disc (IVD) calcification. The length of the branches between two scorer iterations (1, 2), and between each of the three scorers (A, B, C), represents the within-scorer disagreement and between-scorer disagreement, respectively. The scale is based on the number of differing scores out of the 314 IVDs assessed by an individual scorer. Numerical bootstrap values indicate strength. Scale bar = 5 IVD scoring differences

Only a fraction of the IVDs (range, 8 to 19 per dog) were scanned using CT, providing a total of 314 IVDs scored. Overall, a total of 1880 CT scores were obtained from the six scoring iterations, with four scores missing (Fig. 2). The reproducibility of CT for scoring IVD calcification approximated its repeatability, which suggested no scorer effect (Table 1). Indeed, the CT phylogram (Fig. 4) indicated no evidence of clear clusters (all bootstrap values < 70%), confirming a lack of evidence of scorer effect (subjectivity) with CT. The distances between iterations within a scorer and between scorers were similar but long, producing a starfish-shaped tree. This reflects lower within-scorer agreement (repeatability) across all scorers compared to radiography, which subsequently resulted in lower between-scorer agreement (reproducibility).

### Magnetic resonance imaging

**Fig. 5:**
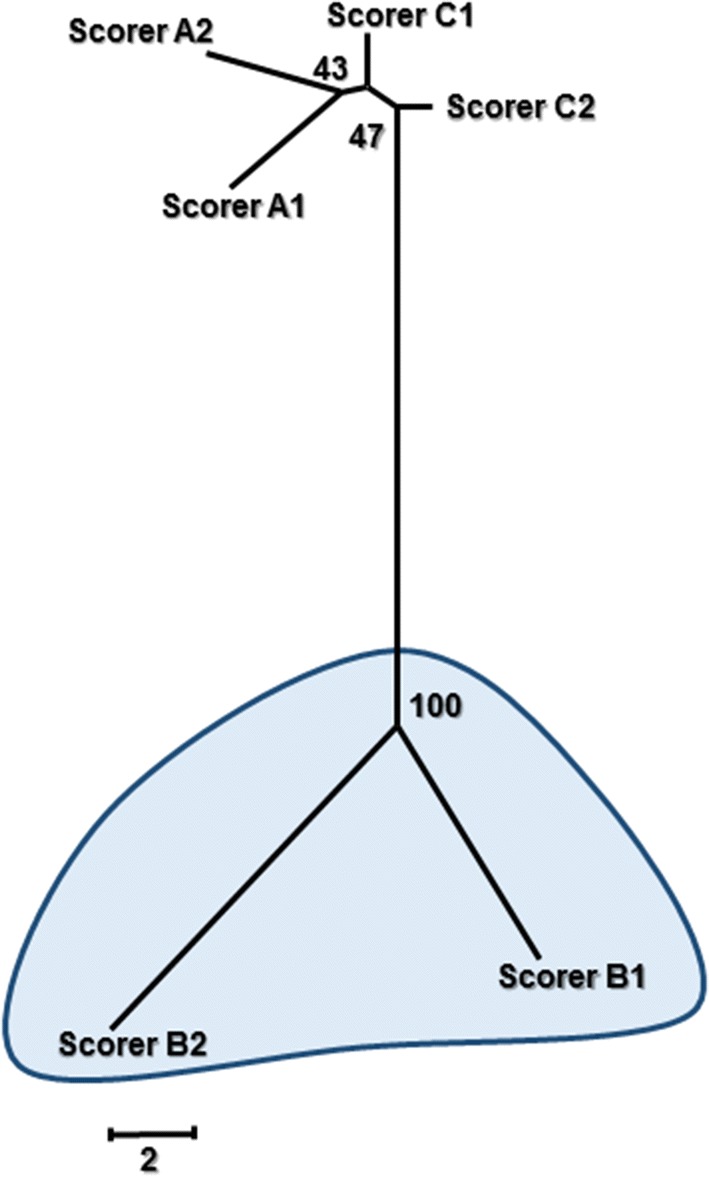
Phylogram demonstrating the agreement within and between scorers for magnetic resonance imaging (MRI) scoring of intervertebral disc (IVD) calcification. The length of the branches between two scorer iterations (1, 2), and between each of the three scorers (A, B, C), represents the within-scorer disagreement and between-scorer disagreement, respectively. The scale is based on the number of differing scores out of the 142 IVDs assessed by an individual scorer. Numerical bootstrap values indicate strength. Scale bar = 2 IVD scoring differences

MRI scans were only available for 11 of the participating dogs and, at most, 14 IVDs per dog were examined. Overall, 142 IVDs were scored with a total of 840 MRI scores obtained from the six scoring iterations. The repeatability of MRI was moderately higher than its reproducibility (Table 1). The MRI phylogram (Fig. 5) identified one strong cluster (bootstrap value 100%) corresponding to scorer B. This suggested that scorer B’s interpretation of MR images was significantly different from the other two scorers (i.e. lower reproducibility for this scorer). The distance between the iterations within scorer B were also clearly longer compared to the iterations within each of the other two scorers, reflecting a lower repeatability for scorer B.

### Comparison of modalities’ precision

Across the three diagnostic imaging modalities, radiography showed the highest repeatability (95.4%) for scoring IVD calcification, and was significantly higher than CT (90.4%) but not significantly higher than MRI (93.8%) (Table 1). There was no significant difference in reproducibility across the three modalities; however, a trend was present with decreasing between-scorer agreement for radiography, followed by CT and then MRI (92.9, 89.4 and 86.4%, respectively).

### Agreement between modalities

Of all three modalities, considerably more IVD calcification was identified by CT (38.8% of all CT scores were positive for calcification) than radiography (8.2% of all radiography scores) and MRI (11.6% of all MRI scores interpreted at Pfirrmann Grade ≥ 3). Regardless of the Pfirrmann grade cut-off used to binarize data into a ‘positive’ or ‘negative’ score for IVD calcification, CT moderately agreed with radiography (approximately 65% agreement) (Table 2). Agreement between MRI and the other two modalities substantially increased at the Pfirrmann grade cut-off ≥ 3 and was highest at the cut-off ≥ 4. However, agreements between modalities at the cut-offs between ≥ 3 and = 5 approximated. At cut-off ≥ 4, MRI and radiography agreed 85.4% of the time (95% CI 80.3–89.3%), while MRI and CT agreed 64.9% of the time (95% CI 56.5–72.4%).

**Table 2:** Agreement between scoring modalities relative to MRI Pfirrmann grade cut-off

Compared modalities	Pfirrmann grade ≥ 1	Pfirrmann grade ≥ 2	Pfirrmann grade ≥ 3	Pfirrmann grade ≥ 4	Pfirrmann grade = 5
Radiography vs. CT	64.2% (58.5–69.4)	64.4% (58.5–69.9)	65.6% (58.0–72.5)	67.0% (59.0–74.2)	67.1% (58.9–74.3)
Radiography vs. MRI	20.1% (16.2–24.6)	46.4% (40.1–52.8)	80.8% (75.1–85.4)	85.4% (80.3–89.3)	83.9% (78.4–88.2)
CT vs. MRI	45.9% (39.9–52.0)	51.1% (44.6–57.5)	62.8% (54.8–70.0)	64.9% (56.5–72.4)	62.8% (54.2–70.7)

Model estimates (95% CI) of pairwise agreement between scoring modalities for each MRI Pfirrmann grade cut-off used to binarize data into a ‘positive’ or ‘negative’ score for intervertebral disc (IVD) calcification

*CT* computed tomography, *MRI* magnetic resonance imaging

## Discussion

Due to the heritability of IVDD and IVD calcification in Dachshunds, selective breeding is important to reduce transmission to offspring [11, 14, 38]. Scoring IVDs for calcification is a reliable predictor of future IVDD development [18], and IVD calcification is currently screened for using conventional radiography. It was predicted that CT and MRI would provide better precision (repeatability and reproducibility) and less subjectivity than radiography when scoring for IVD calcification, as these cross-sectional imaging modalities reduce the confounding effects of anatomic superimposition and provide superior contrast resolution [25]. Despite expectations, neither the repeatability nor reproducibility of CT or MRI was better than the repeatability and reproducibility of radiography. While the repeatability of MRI was similar to that of radiography, the repeatability of CT was significantly less. The reproducibility of both CT and MRI were less than that of radiography, however these were not significantly different. As anticipated for all modalities, estimates of repeatability were higher than estimates of reproducibility, although the two values were very similar for CT. The similar repeatability and reproducibility for CT indicates a lack of individual scorer subjectivity for this modality. Challenges with scoring IVD calcification using CT could have been due to less experience and/or training using this method of screening compared to radiography. Further, CT detected substantially greater overall numbers of calcified IVDs than the other modalities, including discs with smaller total proportion of calcification. This may have led to decreased scorer confidence in assigning a positive or negative score to a given IVD and thus greater variability between scoring iterations.

While the repeatability and reproducibility estimates were similar for both radiography and CT, MRI showed a larger discrepancy between repeatability and reproducibility. The lower level of reproducibility for MRI could be explained by the clear difference in scoring pattern of scorer B compared to scorers A and C (Fig. 5). It is unclear which of the scorers were scoring most correctly (i.e. accurately); regardless, it could be concluded that a degree of difficulty arose when using MRI to screen for IVD calcification, possibly attributable to a lack of experience or training using MRI and the Pfirrmann grading system. On the other hand, our findings are similar to those of others who have evaluated the reliability of the Pfirrmann MRI classification system [30, 33, 39]. When the system was initially evaluated in people, the intra- and inter-observer agreement yielded average kappa scores of 0.88 and 0.77, respectively, with percentage agreements that approximated our results (90.8% and 83.0%, respectively) [33]. A subsequent reliability study was conducted using a modified Pfirrmann grading system, and the intra- and inter-reader agreement remained good but comparatively less (Avg. K scores, 0.86 and 0.66, respectively; Avg. % agreement, 84.9% and 66.8%, respectively) [39]. Variable intra- and inter-observer agreement for scoring canine IVDs for degeneration using the Pfirrmann grading system has been identified (K score range, 0.58 to 0.93) [30, 40]. We chose not to use conventional kappa values because of the recognised limitations of this method including its sensitivity to prevalence [41], which limits direct comparison between our agreement estimates and the kappa results obtained in earlier studies.

The Pfirrmann grading system is based on identifying progressive phases of IVD degeneration [30, 33], not specifically IVD calcification. Although this means that our estimates of agreement for scoring IVD calcification between the different modalities cannot be considered equal, a cut-off Pfirrmann grade ≥ 3 was selected to assign a ‘positive’ score for IVD calcification on MRI. We chose this cut-off as grades of 3, 4 and 5 are assigned to IVDs with changes (reduced MR signal intensity and distinction between nucleus pulposus and annulus fibrosus) that would be expected with more severe IVD degeneration, potentially including some degree of calcification [32, 42]. Further, it is recognised that discriminating between Pfirrmann grades 1 and 2, and between grades 3 and 4, can be challenging and subjective [30, 33, 39], supporting the choice to categorise scores of ≤ 2 as negative and ≥ 3 as positive for calcification. The agreement estimates between modalities at cut-off ≥ 3 approximated those at cut-offs ≥ 4 and = 5 (Table 2).

The recommendation that RDIDC scoring be performed by experts is further supported by the higher precision found in this study for scorers that had specific experience in diagnostic imaging, compared to our prior study using a heterogeneous group of scorers with variable backgrounds [20]. Based on the agreement estimates identified herein, the chance of every IVD within a given dog being scored identically when evaluated twice by the same person (repeatability) is 29.4% (0.954^26^), compared to 12.5% seen previously [20]. Similarly for reproducibility, when a given dog is scored twice by two different scorers the chance of every IVD within that dog being identically scored is 14.7% (0.929^26^), compared to 5.1%. These calculations assume complete independence of individual IVD scoring, which is the worst-case scenario.

Radiography was the only modality of the three to show a clear scorer pattern (i.e. subjectivity), demonstrated as three distinct scoring clusters (Fig. 3). These findings agree with those from our earlier work [20]. The scorer-dependent patterns demonstrated in that study were attributed to scorer differences that might be explained by variation in scoring ability and experience (general practitioner, specialist radiologist, and expert scorer). Comparatively, in the present experiment the scorers had a more similar background and training in diagnostic imaging; therefore, the observed subjectivity is less likely to be attributed to scorer ability but instead may be due to distinct individual scoring styles that could feasibly develop with greater experience. Nevertheless, of the three modalities evaluated, radiography provides consistently higher within- and between-scorer agreement across all 26 potential IVDs, and when the highest level of precision in IVD calcification scoring is desired, radiography should be considered above CT and MRI.

The agreement estimates across the three modalities showed that MRI and radiography agreed more with each other than CT did with either modality. More agreement between radiography and CT might be initially expected as both modalities assess IVD calcification specifically, whereas MRI scoring is based on a wider spectrum of IVD degeneration. However, the lack of modality agreement between radiography and CT, and MRI and CT, is likely due to the substantially larger number of IVD calcifications detected using CT versus the other two modalities. The potential benefits of this higher detection rate using CT need further investigation. Although the relatively good agreement between radiography and low-field MRI (85.7%) could make MRI an acceptable alternative to RDIDC scoring when performed by an individual who is experienced using the Pfirrmann grading system, MRI is substantially more expensive and time consuming to perform than radiography, making it an impractical screening tool for dog breeders.

The results of this study suggest that further insight into the accuracy of each modality is required before considering replacement of radiography with CT or MRI for IVD calcification screening in Dachshunds. As might be expected, the three modalities appeared to detect distinct features of IVD degeneration. While it seems that radiography is the best method of IVD screening in terms of precision, it is suspected that CT is in fact scoring more correctly—that is, CT is more accurate—than radiography and MRI, resulting in the disagreement of CT scores with radiography and MRI. Use of a modified Pfirrmann grading system that is more discriminatory in determining severity of disc degeneration, such as the one developed for elderly people [39], may be warranted in Dachshunds. If CT or MRI were shown to be more accurate than radiography, any gains achieved would need to be balanced with the increased cost, reduced access to the modality in veterinary practice, and overall feasibility for breeders.

Potential limitations of this study might be related to the CT and MRI equipment used, as whole dog spines could not be imaged because of technical limitations, thereby reducing the number of IVDs that were sampled and scored. However, the total number of scores obtained for each modality by the duplicate iterations for each of three scorers was sufficiently high for analysis at the individual IVD level. Analysis of scorer precision at the whole dog level was not performed due to the aforementioned limitations. Further, low-field MRI has known limitations in terms of image quality compared to high-field MRI; nevertheless, the literature indicates that low-field MRI is suitable for grading IVD degeneration in dogs [28, 30–32]. The moderately inconsistent number and position of IVDs imaged by the various modalities in different dogs could have caused human counting error when identifying which IVD was being scored at a given time. However, visual examination of the score summary diagram (Fig. 2) did not identify patterns suggestive of frequent counting or localisation errors.

## Conclusions

While it might be anticipated that more advanced screening modalities, namely CT and MRI, would improve diagnosis of IVD calcification compared to radiographic scoring, this study did not find any improvement in repeatability or reproducibility of those modalities. If an alternative modality were to replace radiography, training in modality-specific scoring should be implemented to increase within- and between-scorer agreement and test robustness. With correct scorer instruction, CT and MRI have the potential to increase the precision of IVD calcification screening. However, it is important to first evaluate the accuracy of CT and MRI to provide appropriate recommendations regarding which, if any, of the alternative modalities should replace radiography for the screening of IVD calcification in Dachshunds.

## Abbreviations

CT: computed tomography
IVD: intervertebral disc
IVDD: intervertebral disc disease
MRI: magnetic resonance imaging
RDIDC: radiographically detectable intervertebral disc calcification
